# Measuring fear evoked by the scariest animal: Czech versions of the Spider Questionnaire and Spider Phobia Beliefs Questionnaire

**DOI:** 10.1186/s12888-021-03672-7

**Published:** 2022-01-06

**Authors:** Jakub Polák, Kristýna Sedláčková, Markéta Janovcová, Šárka Peléšková, Jaroslav Flegr, Barbora Vobrubová, Daniel Frynta, Eva Landová

**Affiliations:** 1 National Institute of Mental Health, Klecany, Czech Republic; 2 Department of Psychology, Faculty of Arts, Charles University, Prague, Czech Republic; 3 Department of Zoology, Faculty of Science, Charles University, Prague, Czech Republic; 4 Department of Philosophy and History of Sciences, Faculty of Science, Charles University, Prague, Czech Republic

**Keywords:** Animal phobia, Arachnophobia, Disease avoidance, DS-R, Fear of spiders, Psychometric analysis, SBQ, SPQ

## Abstract

**Background:**

Although tiny in size and mostly harmless, spiders evoke exceptional fear in a significant part of the population and arachnophobia is one of the most common anxiety disorders with prevalence 2.7–6.1%. Two standard measures have been widely used to reliably assess the emotional and cognitive component of spider fear, the Spider Questionnaire (SPQ) and Spider Phobia Beliefs Questionnaire (SBQ). We aimed to develop and validate their Czech translations, describe distribution of spider fear in the Czech population, and analyse its association with disgust propensity and other sociodemographic characteristics.

**Methods:**

In Phase 1, we developed Czech translations of both questionnaires using a back-translation procedure and then tested their psychometric properties against their English versions in a counterbalanced experimental design using the Mann-Whitney U test and two-sided t-test. In Phase 2, we analysed scores on the Czech SPQ and SBQ on a larger sample. We evaluated the effects of age, gender, level of education, biology background, and association with the assessments of snake fear (i.e. the Snake Questionnaire, SNAQ) and disgust propensity (i.e. the Disgust Scale-Revised, DS-R) using a Spearman correlation, redundancy analysis, and general linear models.

**Results:**

We have demonstrated that the Czech SPQ and SBQ are equivalent to their originals and show excellent test-retest reliability (SPQ: 0.93; SBQ: 0.87–0.90). In total, 398 (10.3%) out of 3863 subjects reached the cut-off point for potential spider phobia. In addition, SPQ and SBQ scores were highly correlated (0.73–0.79), significantly more than with the SNAQ (0.21–0.32) or the DS-R (0.36–0.40). Two multivariate statistical methods revealed a significant association between the gender, age, level of education, biology background, or disgust propensity and the SPQ scores.

**Conclusion:**

The Czech SPQ and SBQ may produce reliable and valid assessments of spider fear, but they must be further psychometrically tested considering the limitation of this study before wider use. We corroborate previous findings that fear of spiders is significantly associated with sociodemographic variables, such as gender, age, or education, as well as with the individual level of disgust propensity.

**Supplementary Information:**

The online version contains supplementary material available at 10.1186/s12888-021-03672-7.

## Background

Arachnophobia, irrational fear of spiders, is one of the most common specific phobias. Based on the literature, arachnophobia affects 2.7–6.1% of people in the general population and is significantly more prevalent among women than men [1]. The average prevalence of spider phobia varies across different countries, ranging from 2.7% in the Netherlands [1], 3.5% in Sweden [2], to even 8.1 and 9.5% in Hungary [3, 4]. In an epidemiological study that surveyed 813 American college students, most of them (34%) reported significant or severe fear of spiders compared to 22% reporting fear of snakes [5]. The exact gender ratio may differ, but it is generally estimated that there may be up to 4 times more spider phobic women [2]. The fact that spiders are a universal human dread is often exploited in the movie industry where many monster archetypes seem to tap into widespread arachnophobia [6].

Spider phobia has a negative impact on the patient’s wellbeing and quality of life [7]. As spiders are frequent cohabitants of humans in their home, they cannot be easily avoided, which puts a strain on the patient’s ability to cope. According to Andrews [8], if panic and phobias are five times less disabling but 20 times more common than schizophrenia, then the total disability attributed to schizophrenia will be a quarter of that due to panic and phobias. It usually puts limits on the individual’s sport activities especially if outdoors [9]. Typically, people with spider phobia express several cognitive biases and distortions as well, manifested in assigning unnatural attributes and unreal intentions to spiders [10]. Despite significant consequences on one’s life, patients suffering from arachnophobia only rarely seek special treatment that is available [11].

Traditionally, psychology research was oriented on the conditioning theory of fear acquisition [12], which tried to explain emergence of spider fear as a result of direct or indirect negative experiences (through vicarious exposure or information transfer) with spiders. Meanwhile, other researchers have taken on the evolutionary perspective claiming that spiders are a prototypical example of ancestral threat to survival and thus, our pre-neolithic ancestors learned to fear them. Subsequently, the tendency to fear and avoid these eight-legged arthropods was genetically fixed and became a permanent phylogenetically rooted module in the human mind. According to this theory, spider fear is biologically prepared, hence the high ratio of people suffering from spider phobia even in the modern world [13, 14].

There is evidence that spider pictures are found faster among fear-irrelevant distractors (e.g., flowers) than vice versa [15]. Higher physiological arousal associated with pupil dilatation to spider pictures (compared to neutral pictures) was observed in 6-month-old infants [16] and event-related brain potentials in 9-month-olds showed increased attention to spiders previously cued with a fearful expression [17].

However, recent studies have shown that spiders, contrary to snakes, do not hold a special status in the visual perception of both humans [18] and primates [19]. Early posterior negativity in the occipital lobes associated with early visual perception of threatening stimuli was greater in response to snake but not spider images [20–22]. Given these results and the fact that venomous spider species capable of delivering a fatal bite were rather scarce throughout the human evolution [23], the potential threat posed by spiders to our ancestors has been repeatedly questioned and has no empirical support [24, 25].

Many recent studies show that spiders elicit a great amount of disgust [26–28]. Specifically, some authors suggest that spiders trigger contamination-based fear [24, 29]. Gerdes and colleagues [25] showed that spiders are unique in eliciting significantly greater fear and disgust than any other arthropod. A disease-avoidance model [30] hypothesize that spider phobia develops from the convergence of the spiders’ disgusting properties and the subjective probability of involuntary physical contact with humans. Indeed, spiders are regarded as highly disgusting by healthy subjects and even more by people with arachnophobia [27], potentially due to their quirky ‘too-many-legs’ body plan. At the same time, they are omnipresent in our homes, often lurking in hidden dark places and capable of fast unpredictable movements. Davey [24] suggested that the disgust-relevant status of the spider resulted from its association with devastating plague pandemics that struck Europe from the Middle Ages onwards. Due to the lack of knowledge of aetiology, the spider seemed like a suitable displaced target.

To quantify fear of spiders, simple self-report measures are used, the most common ones are the Spider Questionnaire (SPQ) [31] and Spider Phobia Beliefs Questionnaire (SBQ) [10]. Especially the former one is widespread in research and clinical practice. It provides a quick evaluation of the respondent’s fear of spiders that may inform initial clinical judgement. Finally, it can also serve as a useful tool in epidemiological studies and when evaluating treatment outcomes. The SPQ has been already translated to several languages including Swedish [32], Dutch [33], or Hungarian [4]. However, a Czech version has been missing and thus, accurate data on prevalence of arachnophobia in the Czech Republic are scarce. Although a shortened 12-item version of the SPQ has been already translated to Czech [34], it is mainly intended as a rapid instrument for screening purposes in large samples. However, in clinical practice and certain research where reliable diagnostics of spider fear is necessary, a more robust and detailed assessment with better psychometric parameters such as higher specificity would be beneficial. Moreover, a majority of people with spider phobia remain undiagnosed as those people do not often seek any specialist help. As for the SBQ, this measure has received only very limited interest since its publication, although it may prove significant contribution to the psychological profile of patients suffering from spider phobia.

Thus, the main aim was to develop Czech translations of the SPQ and SBQ, evaluate their psychometric properties, and describe the prevalence of fear of spiders in the Czech population. In addition, we analysed the effect of basic sociodemographic characteristics on spider fear, such as gender, age, and the level and type of education.

## Phase 1 – translation of the SPQ and SBQ

### Methods

#### Subjects

For this study phase, we recruited a sample of 869 individuals (the age range was 15–88 years [mean age 22.8 ± 0.3]; 613 (70.5%) females). Out of these, 172 subjects completed elementary school, 378 finished high school, and 314 got a university degree, five subjects did not report their education level. The subjects were mostly recruited among high school and college students of natural or social sciences using the quasi-random and snowball sampling. Each participant was approached directly either during a course by one of the co-authors (DF, EL) and other cooperating high-school teachers or outside the class at the school premises (data collected by KS, MJ, ŠP, BV). The rest of participants were staff members of a biology faculty and mental health research institute who were approached directly or by email. In total, 500 subjects had a background in natural sciences compared to 316 subjects with social science education.

#### Procedure

The standardization procedure followed the guidelines for translating and adapting tests set by the International Test Commission [35]; see also [36]. With a permission from the authors, the original SPQ and SBQ were translated from English to Czech independently by two bilingual professionals. These two versions were then checked by a psychologist experienced in test development to identify and resolve potential item discrepancies in the translations. Subsequently, a back-translation to English was performed by another translator unfamiliar with the questionnaire. Another two native English speakers then compared the original and back-translated items to determine whether they were equivalent in meaning. Any substantive differences in particular items were considered and appropriately revised to obtain a translation best corresponding the original instrument.

Next, participants recruited for the study were administered either a pen-and-paper or online version of the SPQ and SBQ in Czech and English on two separate occasions in a counterbalanced experimental design. Thus, a half of the subjects were administered the original English version first, followed by the Czech translation 2 months later. The other half was asked to complete the questionnaires in the reverse order; first in Czech and then in English. The way the participants were divided into these two groups was completely random. We also balanced the order in which the SPQ and SBQ were administered. The selected period of 2 months between each administration is generally recommended when retesting personality questionnaires [37]. It is believed that after this time the subjects can no longer remember their previous answers that could influence the current score, thus the carry over effect is eliminated [38].

Before administering the questionnaires in English, the subjects were asked about their language proficiency and instructed not to complete the measure if they did not feel confident to have well understood the meaning of each item. However, as most of our respondents were high school or university students who have been studying English for several years, their good comprehension of each item may be expected.

#### Questionnaires

##### Spider questionnaire (SPQ)

The SPQ is a 31-item self-report scale to assess the verbal–cognitive component of spider fear. Each item is a fearful or non-fearful statement related to spiders and is rated by the respondent as true or false. The instrument is scored by assigning a ‘1’ to each true response and ‘0’ to each false response, nine items are reversed-scored. A total score (ranging from 0 to 31) is calculated by summing all ‘true’ statements. Psychometric analyses have shown that the SPQ has a high internal consistency as estimated by Kuder-Richardson Formula 20 (e.g., 0.83–0.94 [31] or 0.81–0.89 [32]), excellent test-retest reliability after a year (e.g., r = 0.87 [32]), and satisfactory levels of validity as it can discriminate between people with arachnophobia and healthy controls [32, 33]. There also exists its shortened 12-item version in Czech [34] and Hungarian [3].

##### Spider phobia beliefs questionnaire (SBQ)

The SBQ is a 78-item self-report scale to measure fearful beliefs about spiders and one’s reaction to encountering them. Thus, it has two subscales, first 42 items measure the strength of fearful beliefs related to spiders (spider-related beliefs subscale, SpB), items 43 to 78 then tap into beliefs related to the respondent’s reactions to seeing a spider (self-related beliefs subscale, SrB). Each item is rated on a scale 0–100% reflecting the strength of one’s belief (0 = I do not believe it at all; 100 = I absolutely believe it). A factor analysis revealed 5 spider-related factors: harm, hunter and prey, unpredictability, territory, and multiplication; and 4 self-related factors: panic, paralysis, incubation, and unrestrained behaviour. These factors have good internal consistency (α = 0.68–0.93) and reasonable test-retest reliability (r = 0.57–0.84). Also, both subscale scores have satisfactory test-retest reliability (SpB: r = 0.68; SrB: r = 0.71). Finally, the SBQ demonstrates good concurrent validity as indicated by positive associations with other indices of spider fear and discriminates well between people with arachnophobia and healthy controls [10].

#### Statistical analysis

To verify accuracy of the translation process, we aimed to recruit at least 240 subject who would complete both language versions of the SPQ and SBQ (120 subjects would start with the English original and the other 120 with the Czech translation). This minimum sample size has been identified according to what is common in the current literature on test adaptations [39, 40].

To compare the scores on the original and translated scale, several analyses were performed. First, we checked for the score distribution on the English and Czech scales and conducted the Shapiro-Wilk test (SW) to verify their normal distribution. Due to the non-normal distribution of SPQ and SBQ scores, we employed the Mann-Whitney U test to analyse the effect of administration order on total scores. Then, we continued the analysis with the subjects who filled both language versions. For each respondent, an absolute difference between the English and Czech scores was calculated and then the Mann-Whitney U test was used to verify, whether it was affected by the language of the first test. Subsequently, the Wilcoxon signed-ranked test for related samples was adopted to compare the scores from the original and translated version. Responses on individual items were compared using the McNemar test with a Bonferroni correction (due to the large number of SBQ items, we performed this analysis only for the SPQ).

We also employed a method of statistical equivalence testing, specifically the two one-sided t-test (TOST) [41], to analyse the measurement invariance. An acceptance criterion θ was calculated using the following formula: $\theta =\delta +{s}^{\prime}\left[{t}_{\left(1-\alpha, 2n-2\right)}+{t}_{\left(1-\beta /2,2n-2\right)}\right]\sqrt{\frac{2}{n}}$ (*δ*: the absolute value of the true difference between the groups’ mean values, arbitrarily set to 0; s’: the upper 90% confidence interval (CI) of the standard deviation s; α = β = 0.05). Subsequently, a 90% CI of the difference between the mean total scores from both measures was compared to [−θ; θ] interval and *p*-values of the TOST were calculated.

Finally, a correlation of scores on both language versions was calculated using a.

formula for test-retest reliability (r = $\frac{\mathit{\operatorname{cov}}}{s1\times s2}$, cov: covariate of test-retest, s_1_, s_2_ – standard deviations of the original and translated instrument). All the analyses were performed in the SPSS, version 22 [42] except for the TOST, which was performed in the XLSTAT add-on statistical package for Excel, version 2017.4 [43].

### Results

#### SPQ

Out of 869 respondents recruited for this study, 319 completed the SPQ in both languages (123 subjects did the English version first, while 196 subjects started with the Czech translation), the rest of respondents did not to participate in the second round. Four subjects had an absolute difference of scores on the English and Czech version 10 or higher and these were excluded as outliers from further analyses. The Wilcoxon signed-ranked test revealed a statistically significant difference between total scores on the original and translated instrument with the English scale yielding slightly higher scores (EN: M = 8.16 ± 0.40, CZ: M = 7.81 ± 0.39; *p* = 0.008). Comparison of responses on individual items of both language versions revealed a significant difference at the Bonferroni-corrected level α = < 0.002 on four items (16, 17, 20, and 28).

Based on these results, the studied sample was divided respective to the language order and each data subset was analysed separately. When the original instrument was administered first, it yielded a significantly higher total score (M = 7.93 ± 0.67) compared to retest using the Czech translation (M = 7.39 ± 0.66; *p* <  0.01). Interestingly, the opposite pattern, though less pronounced and statistically nonsignificant, was found when the translated measure was tested first (M = 8.07 ± 0.49) followed by the English original (M = 8.31 ± 0.49; *p* = 0.19).

Despite these outcomes, the TOST confirmed that the two language versions of the SPQ were equivalent in measuring fear of spiders (lower bound: t = − 3.18, *p* <  0.01; upper bound: t = 4.46, p <  0.01). Finally, using the test-retest formula, we found an excellent correlation between scores on the original and translated scale r = 0.93.

#### SBQ

For the SBQ, we collected data from 833 participants, 314 of them completed both the original and translated version (118 subjects had the English SQB first, 196 subjects started with the Czech translation), the rest of respondents chose not to participate in the second round. Six subjects reached an absolute score difference on either of the subscales more than 30 and therefore were excluded from further analyses as outliers.

There was no significant difference in SpB and SrB scores on the English and Czech scale (SpB EN: M = 21.48 ± 1.06; SpB CZ: M = 20.79 ± 1.01; *p* = 0.19; SrB EN: M = 13.04 ± 1.07; SrB CZ: M = 12.70 ± 1.02; *p* = 0.70). Based on the TOST, the two language versions of SBQ can be considered as equivalent (SpB: lower bound: t = − 3.46, *p* <  0.01; upper bound: t = 4.33, p <  0.01; SrB: lower bound: t = − 3.61, p <  0.01; upper bound: t = 4.19, p <  0.01). Finally, both SpB and SrB scores where highly correlated between the English and Czech version (r = 0.87 and r = 0.90, respectively). For more detailed results, please see Additional file 1.

## Phase 2 – psychometric analysis of the Czech SPQ and SBQ

### Methods

#### Subjects

In Phase 2, we aimed to conduct a more detailed psychometric analysis of the Czech SPQ and SBQ. As anxiety associated with spiders may have a significant disgust component, we analysed a relationship between these two instruments and a measure of disgust propensity (Disgust Scale-Revised, DS-R), which can be used as a criterion of convergent validity. We also used a measure of snake fear (Snake Questionnaire, SNAQ) to test for discriminant validity. As shown in the previous phase, scores on the Czech translations did not differ depending on whether the translated scale was administered before or after the original version (except for SpB at *p* = 0.03).

Therefore, for the following analyses, we used data from all the Czech SPQs (*N* = 662) and SBQs (*N* = 634) no matter the order of administration. Additionally, we completed this sample with data from members of a Facebook community including more than 16,000 followers. Several posts published on the Facebook wall were inviting to fill an online battery comprised of three assessments, the SPQ, SNAQ [31] in a Czech translation [40], and the DS-R [44, 45] in a Czech translation [46]. Finally, we also included unpublished questionnaire data from our previous research projects focused, in general, on emotions triggered by various animals.

Doing so, we collected a sample of another 3201 individuals who completed the SPQ. Moreover, 3562 of them also completed the DS-R (out of these, 415 subjects were excluded from further analyses as they did not provide a valid response on one of the catch questions, see below for the test description), while 2585 completed the SNAQ and 399 the SBQ. In this pooled sample of 3863 participants aged 15–88 years (mean age 29.9 ± 0.2), there was a considerably higher proportion of women (2778 vs 1066 men, 9 subjects did not disclose their gender). Most of the participants completed high school (*N* = 2018) or a university degree (*N* = 1489), only a minority have had elementary school as their highest completed education (*N* = 285). Finally, we also gathered information on their field of study and categorized the subjects as having a biology (*N* = 975) or non-biology background (*N* = 2888) as this has been found by previous studies as an important factor affecting animal fears [28, 40].

#### Questionnaires

##### Snake questionnaire (SNAQ)

The SNAQ is a 30-item self-report scale to assess the verbal-cognitive component of snake fear. Each item is a fearful or non-fearful statement related to snakes. Participants rate each item as true or false. The instrument is scored by assigning a “1” to each true response and “0” to each false response, nine items are reversed-scored. A total score (ranging from 0 to 30) calculated by summing all ‘true’ statements serves as a measure of the degree of phobic fear [47, 48]. The SNAQ shows good internal consistency (0.78–0.90 [31] or 0.91 [40]) and excellent test-retest reliability (r = 0.84 [32]) and discriminates well between people with snake phobia and healthy controls [4, 32].

##### Disgust scale – revised (DS-R)

The DS-R is a self-report personality scale to assess individual differences in propensity to disgust. There are 25 disgust elicitor items loading on one of the three factors (core, animal reminder, and contamination-based disgust) and two catch questions (item 12 and 16) to identify those respondents that are not paying attention to the task or do not take it seriously. Each item is rated by the participant on a 5-point Likert scale from 0 (“Strongly disagree/Not disgusting at all“) to 4 (“Strongly agree/Extremely disgusting”). The total score (ranging from 0 to 100) is calculated by summing scores on all the 25 disgust elicitor items but three (item 1, 6, 10) that are reverse scored. Similarly, subscale scores may be calculated. All the participants that do not give valid answers on the catch questions should be dropped. The DS-R demonstrates acceptable Cronbach’s alpha estimates for the overall internal consistency (0.84) and the three subscales (core disgust: 0.74; animal reminder disgust: 0.78; contamination-based disgust: 0.61 [45, 49]).

#### Statistical analysis

For Phase 2, we aimed to gather data from at least 1000 subjects. First, reliability of the Czech SPQ and SBQ was calculated using the split-half method, internal consistency was expressed as the Cronbach’s alpha. To normalize nonlinear score distributions, we applied the McCall area transformation with data continuity adjustment [50]. Using the transformed z-scores we calculated norms for our sample. We also computed a Spearman correlation coefficient between scores on the SPQ, two SBQ subscales (SpB and SrB), and DS-R to demonstrate convergent validity. Based on the literature [33], we would expect a correlation coefficient between the two scales of spider fear at least r = 0.6 and slightly lower between these and the DS-R. Discriminant validity was expressed by a Spearman correlation coefficient between the SPQ/SBQ and SNAQ scores, which should be ideally below r = 0.3.

Next, we employed a General Linear Model (GLM) for a quasibinomial distribution (log-link function) to analyse the effect of sex, age, level of education (categorized as either elementary school, high school, or university), and biology vs. nonbiology education on the SPQ scores. The initial model was further reduced, and the Chi-square criterion was adopted to compare the full and reduced model (ANOVA command).

We have also performed a redundancy analysis (RDA) as implemented in the R package vegan [51] to quantify contribution of the explanatory variables (respondent’s gender, age, highest education, biology background, three subscale scores on the DS-R, and the SNAQ score) on all SPQ item scores. The RDA is a multivariate direct gradient method [52], which extracts and summarizes the variation in a set of response variables and permits to plot both the response and explanatory variables to a space defined by the extracted gradients to detect redundancy (i.e. shared variability). Statistical significance of the gradients was confirmed by permutation tests.

To evaluate effects of the above factors on the SBQ scores, we employed linear models with a square-root transformation improving normality of the data distribution. The full model was further reduced according to the Akaike information criterion (AIC). Finally, we conducted a receiver operating characteristic (ROC) curve analysis and calculated the Youden index (*J* = maximum {sensitivity + specificity - 1} [53] to find the ideal cut-off point on the two SBQ subscales for potential spider phobia as identified by the SPQ score. Calculations were performed in SPSS, version 22 [42] and R, version 3.6.3 [54].

### Results

#### SPQ

**Fig. 1:**
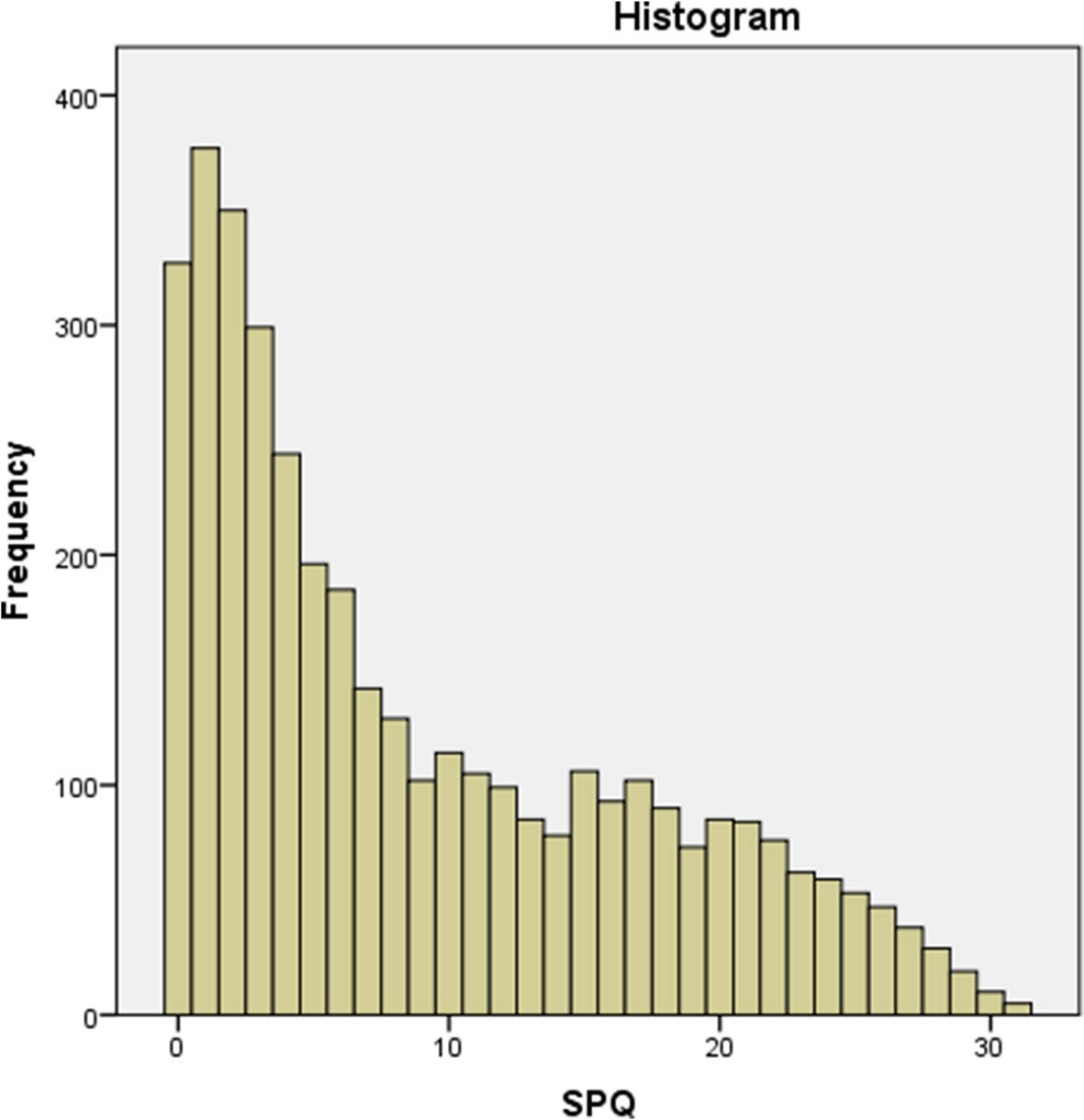
A histogram of the Spider Questionnaire (SPQ) total scores. The graph shows a distribution of the SPQ scores. Mean = 9.02, SD = 8.04; *N* = 3863

Distribution of the Czech SPQ scores significantly deviated from normality (SW = 0.90, df = 3863, *p* <  0.01) with skewness 0.77 ± 0.04 and kurtosis − 0.58 ± 0.08 (see Fig. 1 for raw scores distribution). The translated scale demonstrated high internal consistency (Cronbach’s α = 0.94) and split-half reliability (Guttman split-half coefficient λ = 0.92). The mean score was 9.02 ± 0.13 (SD = 8.04) and median 6.00, standardized McCall transformed scores and respective norms can be found in Additional file 2.

The reduced GLM model revealed a significant effect of gender, age, education level, biology background, core and animal reminder disgust score of the DS-R (all *p*-values < 0.01), and finally the SNAQ score (*p* <  0.01); see Table 1 for SPQ scores according to the gender, education level, and biology background. Contrary to that, the effect of contamination disgust score was not significant (*p* = 0.68); for parameter estimates, see Table 2. For correlation coefficients between the SPQ, SBQ, DS-R, and SNAQ, please see Table 3.

**Table 1:** Descriptive statistics of total scores on the Czech translation of the Spider Questionnaire categorized according to gender, education level, and biology background

		N	Percent	Mean	Median	95% CI of mean
**Overall**		3863	100%	9.02	6.00	8.77–9.28
**Gender**	Men	1072	27.8%	5.44	3.00	5.10–5.79
	Women	2791	72.2%	10.40	8.00	10.09–10.71
**Education level**	Basic school	285	7.4%	10.78	8.00	9.83–11.73
	High school	2018	52.2%	9.46	7.00	9.10–9.81
	University	1489	38.5%	8.01	5.00	7.62–8.40
**Biology background**	No	2888	74.8%	9.39	7.00	9.09–9.69
	Yes	975	25.2%	7.93	5.00	7.47–8.40

**Table 2:** Parameters estimated from the reduced General Linear Model calculating the effect of sociodemographic variables, i.e. the gender, age, education level, biology background, and scores on the Disgust Scale - Revised (its core and animal reminder disgust subscale) and Snake Questionnaire (SNAQ) on the Spider Questionnaire score

Variable	Parameter	p
**Intercept**	−1.91	< 0.01
**Male gender**	−0.48	< 0.01
**Age**	−0.02	< 0.01
**Elementary school**	0.24	0.03
**High school**	0.18	< 0.01
**Biology education**	−0.31	< 0.01
**Core disgust**	0.05	< 0.01
**Animal reminder disgust**	0.02	< 0.01
**SNAQ**	0.01	< 0.01

**Table 3:** Spearman correlation coefficients between the Spider Questionnaire (SPQ), Spider Phobia Beliefs Questionnaire (SBQ), Disgust Scale-Revised (DS-R), and Snake Questionnaire scores (SNAQ). The SBQ has two subscales, the spider-related (SpB) and self-related beliefs (SrB)

	SBQ		DS-R				SNAQ
	SpB	SrB	Total score	Core	Animal rem.	Contam.	Total score
**SPQ**	0.73	0.79	0.40	0.39	0.32	0.22	0.32
**SpB**		0.83	0.39	0.35	0.37	0.19	0.22
**SrB**			0.36	0.32	0.37	0.15	0.21

All coefficients significant at the α = 0.01 level

**Fig. 2:**
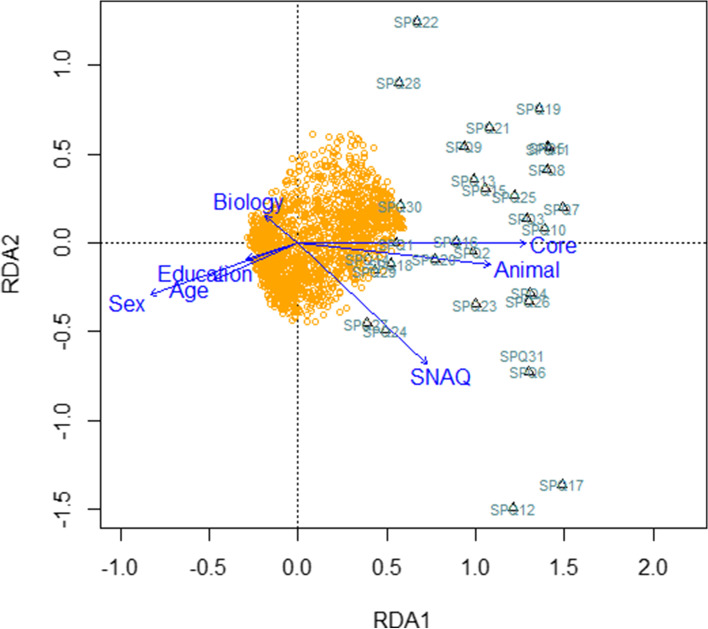
Visualisation of the redundancy analysis (RDA). Redundancy analysis (RDA) of the respondents’ gender (sex), age, level of education, biology background, score on the Snake Questionnaire (SNAQ) and two subscales of the Disgust Scale - Revised, i.e. core and animal reminder disgust as explanatory variables and answers on each item of the Spider Questionnaire (SPQ) as response variables. Yellow circles around the intersection of axes represents individual subjects, black triangles are SPQ items (1–31), which are distributed along the first major axis (RDA1). The second major axis (RDA2) seems to be significantly associated with disgust. Blue arrows signify the direction of explanatory variables’ effect; the longer the arrow, the stronger the effect (i.e. while responses on the SPQ items are positively correlated with the SNAQ, core disgust, and animal reminder disgust score, sociodemographic characteristics on the other hand, such biology background, higher education, older age, and male gender, have the opposite effect). The model explained 10.3% of the full variability

The RDA model of SPQ item scores generated seven constrained axis which explained only 10.3% of the full variability. We then performed a permutation test (number of permutations = 20,000) to confirm the significance of each of the independent variables (constraints) in a sequential (‘type I’) test: gender, F_1,2471_ = 76.13, *p* <  0.001; age, F_1,2471_ = 31.60, *p* <  0.01; education level, F_1,2471_ = 5.76, *p* <  0.01; biology background, F_1,2471_ = 9.74, p <  0.01; core disgust, F_1,2471_ = 91.31, p <  0.01; animal reminder disgust, F_1,2471_ = 10.67, p <  0.01; and SNAQ, F_1,2471_ = 58.85, *p* < 0.01; for visualization of the RDA results see Fig. 2).

Finally, to set a cut-off point for spider phobia and thus be able to estimate its prevalence in the Czech population, we adopted the mean SPQ score 23.76 (SD = 3.80) of 17 subject with spider phobia reported by Fredrikson (1983) and calculated the 95% confidence interval (CI) as 23.76 ± 1.81 ([21.95, 25.57]). We took its lower bound, i.e. SPQ score 22 as a cut-off point for potential spider phobia, which was reached by 398 subjects in our sample representing 10.3% that could be classified as having spider phobia.

#### SBQ

Both the spider-related (SpB) and self-related beliefs score (SrB) on the Czech SBQ significantly deviated from normality (SpB: SW = 0.92, df = 1086, *p* < 0.01, skewness = 0.93 ± 0.07, kurtosis = 0.34 ± 0.15; SrB: SW = 0.79, df = 1086, *p* < 0.01, skewness = 1.48 ± 0.07, kurtosis = 1.57 ± 0.15 (for raw score distributions of SpB and SrB subscale, please see Additional files 3 and 4). The translated scale showed excellent reliability, expressed either through internal consistency (Cronbach’s α = 0.98) or Guttman split-half coefficient (λ = 0.91). The mean SpB score was 24.65 ± 0.55 (SD = 18.16), mean SrB score 15.68 ± 0.61 (SD = 19.96).

Based on the reduced linear models, the SpB score was significantly affected only by the biology background (F_(1, 392)_ = 10.62, *p* = 0.001) an scores on core (F_(1, 392)_ = 9.29, *p* = 0.002) and animal reminder disgust (F_(1, 392)_ = 55.84, *p* < 0.01); the model explained 16.02% of total variability. The SrB score was only affected by gender (F_(1, 392)_ = 33.15, *p* < 0.001) and animal reminder disgust (F_(1, 392)_ = 43.95, p < 0.01); the model explained 16.96% of total variability; for parameter estimates, see Table 4. For SpB and SrB scores according to the gender, education level, and biology background, see Table 5.

**Table 4:** Parameters estimated from the reduced linear model calculating the effect of sociodemographic variables, i.e. gender, age, education level, biology background, and scores on the Disgust Scale - Revised (only its core and animal reminder disgust subscale) and Snake Questionnaire (SNAQ) on two subscale scores of the Spiders Phobia Beliefs Questionnaire, the spider-related (SpB) and self-related (SrB) score. Only significant effects are reported

Variable	SpB		SrB	
	Parameter	p	Parameter	p
**Intercept**	2.96	< 0.01	1.23	< 0.01
**Male gender**	–	< 0.01	−0.89	< 0.01
**Biology education**	−0.37	0.04	–	–
**Core disgust**	0.05	< 0.01	–	–
**Animal reminder disgust**	0.07	< 0.01	0.10	< 0.01

**Table 5:** Descriptive statistics of total scores on the Czech translation of the Spider Phobia Beliefs Questionnaire categorized according to the gender, education level, and biology background. The assessment is divided in two subscales, the spider-related (SpB) and self-related beliefs (SrB)

		N	Percent	SpB		SrB	
				Mean	95% CI	Mean	95% CI
**Overall**		1086	100%	24.65	23.57–25.73	15.68	14.49–16.87
**Sex**	Men	291	26.8%	17.60	16.08–19.12	7.20	5.75–8.65
	Women	795	73.2%	27.23	25.91–28.56	18.78	17.30–20.26
**Education level**	Basic school	120	11.3%	27.23	23.59–30.87	17.55	13.81–21.30
	High school	446	42.0%	25.30	23.53–27.07	16.22	14.25–18.20
	University	497	46.7%	23.34	21.87–24.81	14.55	12.94–16.17
**Biology background**	No	481	44.3%	28.47	26.67–30.27	19.06	17.13–20.99
	Yes	605	55.7%	21.62	20.35–22.88	12.99	11.54–14.44

Finally, based on the previously found threshold of SPQ score 22 and higher, we identified 98 subjects with potential spider phobia in the subsample of subjects who also completed the SBQ. By calculating the Youden index for each coordinate of the ROC curve, we found a cut-off point of 32.64 on the SpB subscale, which corresponds to sensitivity 0.87 and specificity 0.75; J = 0.62. The area under curve (AUC) was 0.87. For the SrB subscale, we found a cut-off point of 25.79, which corresponds to sensitivity 0.847 and specificity 0.81; J = 0.66. The AUC for this ROC curve was 0.87.

## Discussion

In the first part, we demonstrated using different statistical approaches that the Czech translation of both scales measuring spider fear (SPQ and SBQ), can be considered as equivalent to their English original versions. Despite the large number of items, 31 in the case of the SPQ and 78 for the SBQ, the level of similarity was excellent.

In case of the SPQ, responses on four items were statistically different when comparing the original and translated questionnaire. Correlation between the total scores calculated as test-retest reliability was exceptionally high (r = 0.93). This result is even above the range of 0.7–0.9 recommended for test-retest reliability of psychological assessment [55] and higher than test-retest reliability 0.87 reported by Fredrikson [32]. Moreover, both language versions were shown to be statistically equivalent. Very similar results showing high score correlation and statistical equivalence were also found for the two subscales of the SBQ. Thus, our results clearly demonstrate that despite a relatively long delay between both administrations, which exceeded the usually advised period of 1 month, responses on both measures of spider fear are relatively consistent. The adapted Czech translations provide reliable scores, which correlate significantly with the original instruments.

When we split the data sample according to the language tested first, we found slightly higher scores on the English SPQ but only when this one was completed prior to its translation. This is fairly a common phenomenon within studies of translated psychometric tests. Interestingly, a very similar trend of over-scoring of the Czechs when completing the assessment in English has been recently found in a psychometric study of the SNAQ, a self-report measure of snake fear [40] and the DS-R, a measure of disgust propensity [46]. It might be attributed to minor comprehension difficulties, although the overall level of English proficiency among our subject was high. Or, it is also possible that the translation process has slightly shifted meaning of the items despite a thorough back-translation procedure used in translation development. Finally, there might be an effect of language on individual tendencies to self-report, which would be worth studying further.

We have also shown that scores on the SPQ and SBQ were highly correlated, which provides evidence for convergent validity. The correlation coefficients between the SPQ score and two subscale scores of the SBQ were even higher than the expected value It shows that both assessments measuring slightly different components of spider fear can satisfactorily substitute one another. As disgust plays an important role in development of spider phobia [26, 56], correlation between the studied scales of spider fear and the DS-R can be considered as an additional criterion of convergent validity. In this study, the correlation coefficient between the scales of spider fear and the DS-R total score reached the expected value of 0.4. However, it was slightly weaker for the specific disgust subscales, the highest correlation was with core disgust, contrary to contamination-based disgust that correlated the least with the SPQ and SBQ.

As expected prior to the analysis, the correlation coefficient was one of the lowest (r = 0.2–0.3; see Tab. 3). Interestingly though, even weaker correlation was found between the SPQ/SBQ scores and contamination-based disgust score of the DS-R. First, it should be noted that based on a factor analysis of the DS-R, contamination-based disgust as a separate factor has received the weakest statistical support of all three subscales [45, 46]. Second, we could hypothesize that apart from potential comorbidity of snake and spider fear in a certain part of the population, both the SPQ and SNAQ might capture some component of general fearfulness reflected in the correlation coefficient.

In line with the previous research on spider fear, our results provide strong evidence that personal characteristics such as the gender, age, level of education, or biological knowledge serve as significant protective factors against developing spider phobia [2–4, 28, 57]. Specifically, men, older subjects, and people with high school or college education, especially in biology, hence having more information about spiders, score considerably lower compared with women and younger individuals with non-biological or lower education. It should be noted, however, that the association between biology education and fear of spiders is not straightforward. Two completely opposing hypotheses might explain such an outcome. Enrolling for biology degree might either serve as a preliminary selection factor as people with higher fear of spiders simply try to avoid their feared object and thus do not choose to study biology. Or, biology courses may provide knowledge and a certain level of exposure to spiders, which may consequently have a therapeutic effect [58]. As the design of our study does not allow us to resolve this issue, more research is warranted.

The level of spider fear in the Czech sample as measured by the mean SPQ score almost exactly matches the one found by Zsido [4] in Hungary (men: 7.08; women: 10.99) and is very close to the original data in the US [31] (men: 5.57–6.66; women: 8.82–12.43). On the contrary, it is considerably higher than in the Swedish sample [32] (men: 3.80; women: 5.02), although the sample size there was much lower than in our study. Furthermore, score 22 identified based on Fredrikson’s study [32] as a cut-off point for clinically relevant fear of spiders was reached by 10.3% of subjects. Such a proportion of subjects who might suffer from spider phobia is exceptionally high when compared to the literature. For example, Oosterink and colleagues [1] by surveying nearly 2000 Dutch adults found prevalence of spider phobia 2.7%. Similar results were reported from Sweden [2] where 1.2% of men and 5.6% of women were identified as having spider phobia. On the other hand, data from the Hungarian translation of the SPQ demonstrated that 9.5% participants might classify for spider phobia [4] and 14% of American college students claimed to suffer from severe fear of spiders [5].

Such variability in prevalence of spider phobia is counterintuitive should we accept the evolutionary hypothesis of genetically fixed fear acquisition, because in that case, fear of spiders would have been comparable across the world. Moreover, varying prevalence does not reflect diversity and abundance of dangerous spider species in different areas and can neither be explained by cultural differences as these are all Western countries with the similar arachnofauna. For the same reason, it is unlikely that for example the Swedes [2] or Dutchmen [1] would be exposed to spiders significantly more often than the Hungarians, which would lead to fear inoculation. We might therefore hypothesize that it is mainly differences in the sample characteristics and employed diagnostic criteria (cut-off points) that account for the observed variance in prevalence of spider phobia.

The prevalence figures reported above make the spider one of the scariest animals. Given the lack of evolutionary-relevant threat posed by spiders [59, 60], Davey [24] proposed a hypothesis that the spider’s potential to elicit fear in a significant part of the population is associated with its disgusting properties. He claimed that spiders are feared mainly because in the past they served as a displaced target for many inexplicable diseases causing devastating epidemics including the plaque that struck the European and Asian populations from the tenth century onwards and led to millions of deaths. This was due to spiders being found living in the vicinity of the real disease vector, the black rat. In that case, fear of spiders would not be a universal biologically prepared fear, but rather a phenomenon specific to the cultural and historical context of Europe and Middle East.

Although there is a consensus that spider phobia has a strong disgust component, Davey’s hypothesis [24] is still controversial. To date, there has been no direct supportive evidence. A cross-cultural study of animal fears in seven countries showed that fear of spiders was significantly lower in India than in the Western world (Europe and the US), but it was also lower in Holland than Hong Kong or Japan, while Japanese subjects reported higher fear of spiders compared with Brits and Americans [61]. If the ‘plague’ hypothesis was right, spider fear would need to be transmitted cultural and acquired through social learning, which does not explain why for six-months-old infants without any knowledge or prior experience respond to spider images with increased arousal [16].

Finally, our data do not corroborate another suggestion of Davey [24] that spiders might also be feared because of their supposed contamination properties (ability to absorbed poisons in their environment from plants and transfer them through contact). The effect of contamination-based disgust score was significant in none of the statistical methods applied.

### Limitations

Some limitations of this study should be noted. First, although we expected our subjects to have a good command of English based on their curriculum, we have not tested their language skills prior to completing the original scales. Therefore, we cannot completely rule out the possibility that lower comprehension of some participants might have affected their scores. Second, we did not include any test of construct validity of the SPQ and SBQ, for example by conducting a confirmatory factor analysis, because this should be the focus of our following study. Third, there was 415 subjects who were excluded from Phase 2 due to failing on at least one of the two validity items of the DS-R. Although this may seem like a high proportion (corresponding to more than 10% of all subjects who completed the scale), they had to be dropped out to ensure data quality. And finally, fourth, although we succeeded to gather a large sample size as compared with similar published research, we have not performed a formal power analysis prior to data collection to determine the required minimum number of subjects. We rather set our goals according to the common practice in test adaptation and psychometric analyses literature.

## Conclusion

It has been demonstrated that total scores on the original and translated assessments of spider fear (SPQ and SBQ) are highly correlated and the two Czech translations can be considered as statistically equivalent to their original versions. We also provide satisfactory evidence for discriminant and convergent validity of the SPQ and SBQ through correlations with other assessments. However, we have not tested their construct validity yet. This should be addressed before the instruments can be widely used, which will be the focus of our next study (Polák et al., in prep).

On average, male gender, older age, higher education, biology background, and lower tendency to respond with disgust to various stimuli are all associated with lower fear of spiders. Disgust propensity (DS-R), especially its core and animal-reminder subscale, significantly affected also self- and spider-related beliefs as measured by the SBQ. In conclusion, these data corroborate previous literature and provide a strong support for the association between disgust and spider phobia.

## Supplementary Information

**Additional file 1.** Word document - detailed results of Phase 1.

**Additional file 2.** Excel data file - raw and transformed Spider Questionnaire scores with norms.

**Additional file 3.** TIFF file - raw score distribution on the spider-related subscale (SpB) of the Spider Phobia Beliefs Scale (SBQ).

**Additional file 4.** TIFF file - raw score distribution on the self-related subscale (SrB) of the Spider Phobia Beliefs Scale (SBQ).

**Additional file 5.** Excel data file - all the original data analysed in this article.

## Data Availability

The dataset supporting the conclusions of this article is included within the article as Additional file 5.
