# Supplementary material for: Measuring fear evoked by the scariest animal: Czech versions of the Spider Questionnaire and Spider Phobia Beliefs Questionnaire
Source: BMC Psychiatry. 2022 Jan 6;22:18. doi: 10.1186/s12888-021-03672-7 (PMC8740501; doi:10.1186/s12888-021-03672-7)
Supplement: Supplementary file 1 — Additional file 1. Word document - detailed results of Phase 1. [file 12888_2021_3672_MOESM1_ESM.docx]

**Phase 1 - detailed results**

***SPQ***

Out of 869 respondents recruited for this study, 319 completed the SPQ in both languages (123 subjects did the original version first, while 196 subjects started with the translated scale), the rest of respondents chose not to participate in the second round (207 completed only the English version, while 343 did only the Czech one, i.e. we gathered data on 526 English and 662 Czech SPQs). Scores on both the original and translated scale significantly deviated from normality (EN: skewness 0.638, kurtosis -0.795, SW = 0.908, df = 526, p < 0.001; CZ: skewness 0.943, kurtosis -0.017, SW = 0.892, df = 662, p < 0.001). We adopted the Mann-Whitney U test to compare the SPQ scores according to the order of administration for both languages separately. People who completed the original version first scored significantly higher compared to those who did it in the second round (EN first: M = 10.36 ± 0.46, SD = 8.43; EN second: M = 8.46 ± 0.49, SD = 6.87; p = 0.028). There was a similar trend, though nonsignificant, for the Czech translation to yield slightly higher scores on the first compared to second administration (CZ first: M = 8.09 ± 0.30, SD = 6.91; CZ second: M = 7.37 ± 0.65, SD = 7.24; p = 0.140).

From those who completed both language versions, four subjects had an absolute difference of scores on the English and Czech version 10 or higher and these were excluded as outliers from further analyses. According to the Mann-Whitney U test, the mean absolute score difference (∆M = 1.94 ± 0.11, SD = 1.86) was unaffected by the language of the first administration (EN first: ∆M = 1.82 ± 0.16, SD = 1.71; CZ first: ∆M = 2.01 ± 0.14, SD = 1.95; p = 0.568). However, when directly comparing the total scores on both language versions, a statistically significant difference was found with the English scale yielding slightly higher scores (EN: M = 8.16 ± 0.40, SD = 7.03; CZ: M = 7.81 ± 0.39, SD = 6.97; p = 0.008). Comparison of responses on individual items of both language versions revealed a significant difference at the Bonferroni-corrected level α = < 0.002 on 4 items (16, 17, 20, and 28).

Based on these results, the studied sample was divided respective to the language order and each data subset was analysed separately. When the original instrument was administered first, it yielded a significantly higher total score (*M* = 7.93 ± 0.67, *SD* = 7.40) compared to the retest using the Czech translation (*M* = 7.39 ± 0.66, *SD* = 7.27); p = 0.008. Interestingly, the opposite pattern, though less pronounced and statistically nonsignificant, was found when the translated measure was tested first (*M* = 8.07 ± 0.49, *SD* = 6.78) followed by the English original (*M* = 8.31 ± 0.49, *SD* = 6.79); p = 0.193.

Despite these outcomes, the TOST confirmed that the two language versions of the SPQ were equivalent in measuring fear of spiders. The 90% CI of the difference in mean total scores (-0.56; 1.27) was completely contained within the interval of acceptance criterion (-2.13; 2.13). This was supported by the left and right one-sided t-test on the lower and upper bound; t = -3.18, df = 628, p = 0.001 and t = 4.46, p < 0.001, respectively. Finally, using the test-retest formula, we found an excellent correlation between scores on the original and translated scale r = 0.928.

***SBQ***

For the SBQ, we collected data from 833 participants, 314 of them completed both the original and translated version (118 subjects had the English SQB first, 196 subjects started with the Czech translation), the rest of respondents chose not to participate in the second round (200 completed the scale only in English and 319 only in Czech, thus overall, we gathered 514 English and 633 Czech SBQs). Subscale scores (SpB and SrB) on both the original and translated scale significantly deviated from normality (EN SpB: skewness 0.945, kurtosis 0.181, SW = 0.909, df = 514, p < 0.001; EN SrB: skewness 1.372, kurtosis 0.992, SW = 0.781, df = 514, p < 0.001; CZ SpB: skewness 0.982, kurtosis 0.439, SW = 0.914, df = 633, p < 0.001; CZ SrB: skewness 1.505, kurtosis 1.677, SW = 0.781, df = 633, p < 0.001).

The Mann-Whitney U test showed that when the original scale was administered first, it yielded higher scores than when it was completed at the second administration (EN SpB first: M = 26.62 ± 1.13, SD = 20.13; EN SpB second: M = 20.90 ± 1.40, SD = 19.65; p < 0.001; EN SrB first: M = 18.46 ± 1.26, SD = 22.53; EN SrB second: M = 12.78 ± 1.27, SD = 17.72; p = 0.013). Similarly, scores on the Czech SBQ tended to be higher at the first administration, although the difference was statistically significant only for the SpB subscale (SpB CZ first: M = 22.96 ± 0.75, SD = 17.07; SpB CZ second: M = 20.85 ± 1.75, SD = 18.97; p = 0.031; SrB CZ first: M = 13.81 ± 0.77, SD = 17.46; SrB CZ second: M = 13.22 ± 1.73, SD = 18.79; p = 0.156).

Out of 314 respondents who completed the SBQ in both languages, six subjects reached an absolute score difference on either of the subscales more than 30 and therefore were excluded from further analyses as outliers. The mean absolute difference in SpB and SrB scores between both language versions (SpB: ∆M = 6.70 ± 0.36, SD = 6.31; SrB: ∆M = 4.72 ± 0.37, SD = 6.57) was unaffected by the language of the first administration (SpB EN first: ∆M = 7.06 ± 0.56, SD = 6.10; SpB CZ first: ∆M = 6.49 ± 0.47, SD = 6.44; p = 0.144; SrB EN first: ∆M = 5.26 ± 0.68, SD = 7.31; SrB CZ first: ∆M = 4.39 ± 0.44, SD = 6.07; p = 0.599). Furthermore, there was no significant difference in SpB and SrB scores on the English and Czech scale (SpB EN: M = 21.48 ± 1.06, SD = 18.61; SpB CZ: M = 20.79 ± 1.01, SD = 17.71; p = 0.186; SrB EN: M = 13.04 ± 1.07, SD = 18.70; SrB CZ: M = 12.70 ± 1.02, SD = 17.87; p = 0.704).

Based on the TOST, the two language versions of SBQ can be considered equivalent. The 90% CI for the difference in mean SpB scores (-1.77; 3.04) was completely contained within the interval of acceptance criterion (-5.68; 5.68) as well as was the 90% CI for the difference in mean SrB scores (-1.99; 2.85) contained within the interval of acceptance criterion (-5.73; 5.73). For both subscales, this was supported by the left and right one-sided t-test on the lower and upper bound (SpB: t = -3.46, df = 626, p < 0.001 and t = 4.33, p < 0.001; SrB: t = -3.61, df = 626, p < 0.001 and t = 4.19, p < 0.001, respectively). Finally, both SpB and SrB scores where highly correlated between the English and Czech version (r = 0.873 and r = 0.903, respectively).
