# Supplementary material for: Measuring fear evoked by the scariest animal: Czech versions of the Spider Questionnaire and Spider Phobia Beliefs Questionnaire
Source: BMC Psychiatry. 2022 Jan 6;22:18. doi: 10.1186/s12888-021-03672-7 (PMC8740501; doi:10.1186/s12888-021-03672-7)
Supplement: Supplementary file 2 — Additional file 2. Excel data file - raw and transformed Spider Questionnaire scores with norms. [file 12888_2021_3672_MOESM2_ESM.docx]

**Additional file 2.** Raw and transformed Spider Questionnaire scores with norms; McCall area transformation with a continuity correction (cumulative frequency) was applied.

| **Raw score** | **Absolute frequency** | **Relative frequency** | **Cumulative frequency** | **Z-score** | **Percentile** |
| --- | --- | --- | --- | --- | --- |
| 0 | 327 | 0.0846 | 0.0846 | -1.724 | 4.2 |
| 1 | 377 | 0.0976 | 0.1822 | -1.110 | 13.3 |
| 2 | 350 | 0.0906 | 0.2728 | -0.747 | 22.8 |
| 3 | 299 | 0.0774 | 0.3502 | -0.491 | 31.2 |
| 4 | 244 | 0.0632 | 0.4134 | -0.301 | 38.2 |
| 5 | 196 | 0.0507 | 0.4641 | -0.154 | 43.9 |
| 6 | 185 | 0.0479 | 0.5120 | -0.030 | 48.8 |
| 7 | 142 | 0.0368 | 0.5488 | 0.076 | 53.0 |
| 8 | 129 | 0.0334 | 0.5822 | 0.165 | 56.5 |
| 9 | 102 | 0.0264 | 0.6086 | 0.241 | 59.5 |
| 10 | 114 | 0.0295 | 0.6381 | 0.314 | 62.3 |
| 11 | 105 | 0.0272 | 0.6653 | 0.390 | 65.2 |
| 12 | 99 | 0.0256 | 0.6909 | 0.462 | 67.8 |
| 13 | 85 | 0.0220 | 0.7129 | 0.530 | 70.2 |
| 14 | 78 | 0.0202 | 0.7331 | 0.592 | 72.3 |
| 15 | 106 | 0.0274 | 0.7605 | 0.665 | 74.7 |
| 16 | 93 | 0.0241 | 0.7846 | 0.747 | 77.3 |
| 17 | 102 | 0.0264 | 0.8110 | 0.834 | 79.8 |
| 18 | 90 | 0.0233 | 0.8343 | 0.926 | 82.3 |
| 19 | 73 | 0.0189 | 0.8532 | 1.010 | 84.4 |
| 20 | 85 | 0.0220 | 0.8752 | 1.099 | 86.4 |
| 21 | 84 | 0.0217 | 0.8970 | 1.206 | 88.6 |
| 22 | 76 | 0.0197 | 0.9166 | 1.321 | 90.7 |
| 23 | 62 | 0.0160 | 0.9327 | 1.437 | 92.5 |
| 24 | 59 | 0.0153 | 0.9480 | 1.558 | 94.0 |
| 25 | 53 | 0.0137 | 0.9617 | 1.694 | 95.5 |
| 26 | 47 | 0.0122 | 0.9739 | 1.849 | 96.8 |
| 27 | 38 | 0.0098 | 0.9837 | 2.029 | 97.9 |
| 28 | 29 | 0.0075 | 0.9912 | 2.240 | 98.7 |
| 29 | 19 | 0.0049 | 0.9961 | 2.493 | 99.4 |
| 30 | 10 | 0.0026 | 0.9987 | 2.796 | 99.7 |
| 31 | 5 | 0.0013 | 1.0000 | 3.217 | 99.9 |
