# Supplementary figures and images for: Measuring fear evoked by the scariest animal: Czech versions of the Spider Questionnaire and Spider Phobia Beliefs Questionnaire
Source: BMC Psychiatry. 2022 Jan 6;22:18. doi: 10.1186/s12888-021-03672-7 (PMC8740501; doi:10.1186/s12888-021-03672-7)

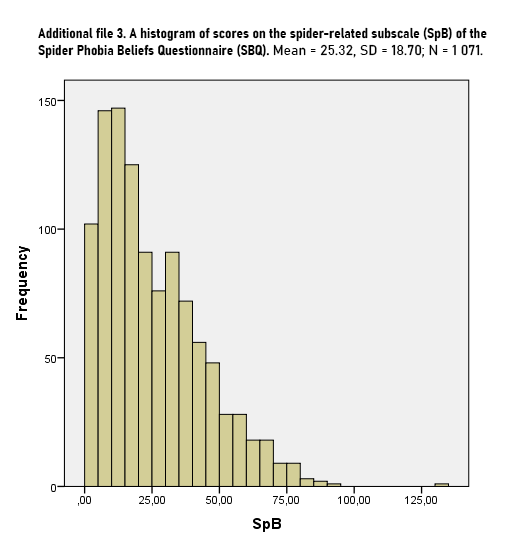

Supplement: Supplementary file 3 — Additional file 3. TIFF file - raw score distribution on the spider-related subscale (SpB) of the Spider Phobia Beliefs Scale (SBQ). [file 12888_2021_3672_MOESM3_ESM.tif]

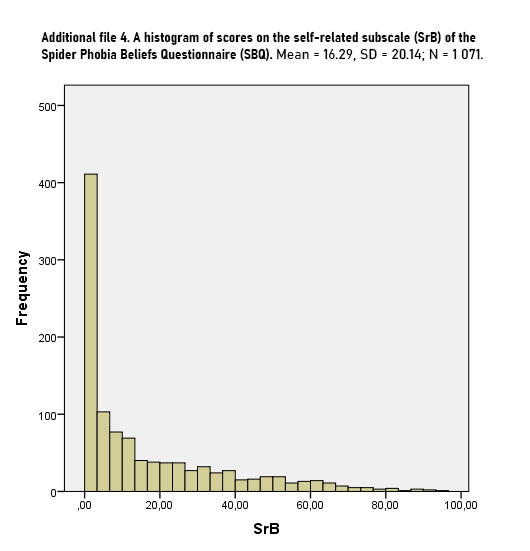

Supplement: Supplementary file 4 — Additional file 4. TIFF file - raw score distribution on the self-related subscale (SrB) of the Spider Phobia Beliefs Scale (SBQ). [file 12888_2021_3672_MOESM4_ESM.tif]
